# CsMYB15 positively regulates *Cs4CL2*-mediated lignin biosynthesis during juice sac granulation in navel orange

**DOI:** 10.3389/fpls.2023.1223820

**Published:** 2023-06-30

**Authors:** Fang Song, Zixuan Li, Ce Wang, Yingchun Jiang, Zhijing Wang, Ligang He, Xiaofang Ma, Yu Zhang, Xin Song, Jihong Liu, Liming Wu

**Affiliations:** ^1^ Hubei Key Laboratory of Germplasm Innovation and Utilization of Fruit Trees, Institute of Fruit and Tea, Hubei Academy of Agricultural Science, Wuhan, China; ^2^ Hubei Hongshan Laboratory, Wuhan, China; ^3^ College of Horticulture and Forestry Sciences, Huazhong Agricultural University, Wuhan, China

**Keywords:** ‘Lane late’ navel orange, granulation, transcription factor, CsMYB15, lignin biosynthesis

## Abstract

‘Lane Late’, a late-maturing navel orange cultivar, is mainly distributed in the Three Gorges Reservoir area, which matures in the late March of the next year and needs overwintering cultivation. Citrus fruit granulation is a physiological disorder, which is characterized by lignification and dehydration of juice sac cells, seriously affecting the commercial value of citrus fruits. The pre-harvest granulation of late-maturing navel orange is main caused by low temperature in the winter, but its mechanism and regulation pattern remain unclear. In this study, a SG2-type R2R3-MYB transcription factor, *CsMYB15*, was identified from *Citrus sinensis*, which was significantly induced by both juice sac granulation and low temperature treatment. Subcellular localization analysis and transcriptional activation assay revealed that CsMYB15 protein was localized to the nucleus, and it exhibited transcriptional activation activity in yeast. Over-expression of *CsMYB15* by stable transformation in navel orange calli and transient transformation in kumquat fruits and navel orange juice sacs significantly increased lignin content in the transgenic lines. Further, Yeast one hybrid, EMSA, and LUC assays demonstrated that CsMYB15 directly bound to the *Cs4CL2* promoter and activated its expression, thereby causing a high accumulation of lignin in citrus. Taken together, these results elucidated the biological function of CsMYB15 in regulating *Cs4CL2*-mediated lignin biosynthesis, and provided novel insight into the transcriptional regulation mechanism underlying the juice sac granulation of late-maturing navel orange.

## 1 Introduction

Citrus is the largest fruit crop in the world, and it is valued due to its abundant functional components beneficial to human health. However, granulation (also known as crystallization) has been a serious problem in the fruit juice sacs of many citrus varieties, including sweet orange (*Citrus sinensis*) (Jia et al., 2018; Jia et al., 2019), mandarin (*Citrus reticulata*) (Yao et al., 2018), grapefruit (*Citrus paradisi*) (Burns and Albrigo, 1998), and pummelo (*Citrus grandis*) (Wu et al., 2014; Shi et al., 2020; Chen et al., 2021). Granulation is a physiological disorder, which causes a reduction in juice, sugar, acid, and flavor substances of citrus fruits, leaving behind the dry, tough, and colorless granulated juice sacs (Theanjumpol et al., 2019; Chen et al., 2021). Granulation usually occurs in either post-harvest storage stage of most citrus varieties or on-tree stage of late-ripening citrus varieties (Burns and Albrigo, 1998; Wu et al., 2014; Chen et al., 2021). Due to the serious damage of granulation to citrus industry, numerous studies have been undertaken to investigate the physiological and molecular mechanisms of citrus granulation, as well as its prevention and control measures (Burns and Albrigo, 1998; Wang et al., 2014; Wu et al., 2014; Yao et al., 2018; Theanjumpol et al., 2019; Shi et al., 2020; Chen et al., 2021). However, most studies are focused on the granulation during post-harvest storage stage, the studies about the juice sac granulation during pre-harvest on-tree ripening stage are very limited (Wu et al., 2020).

Previous studies have revealed that citrus fruit granulation is correlated with lignin deposition, and lignin contents are observed to increase in the granulated juice sacs in many citrus varieties such as mandarin (Sharma et al., 2015; Yao et al., 2018), sweet orange (Jia et al., 2018; Wu et al., 2020; Zhang et al., 2021), and pummelo (Wu et al., 2014; Shi et al., 2020). Lignin, an important component of secondary cell wall, has been reported to play a critical role in citrus granulation process (Jia et al., 2018; Shi et al., 2020). In plants, the lignin biosynthetic pathway involves a series of sequential enzymes, including phenylalanine ammonia lyase (PAL), cinnamate 4-hydroxylase (C4H), 4-coumarate CoA ligase (4CL), shikimate/quinate hydroxy cinnamoyl transferase (HCT), p-coumarate 3-hydroxylase (C3H), caffeic acid O-methyltransferase (COMT), cinnamoyl CoA reductase (CCR), cinnamyl alcohol dehydrogenase (CAD), peroxidase (POD), laccase (LAC), and ferulate 5-hydroxylase (F5H) (Shi et al., 2020; Xie et al., 2020).

The NAC (NAM-ATAF-CUC)-MYB (myeloblastosis)-mediated gene regulatory network responsible for lignin biosynthesis has been well studied in plants (Liu et al., 2015; Ohtani and Demura, 2019). In this regulatory hierarchy, AtNST1 (NAC secondary wall thickening promoting factor 1), AtSND1 (NAC domain protein 1), and AtVND6/7 (vascular-related NAC domain 6/7) have been identified as upstream key regulators for lignin biosynthesis and secondary cell wall formation (Kubo et al., 2005; Mitsuda et al., 2007; Geng et al., 2019). The R2R3-MYB domain MYB transcription factors (TFs) AtMYB46 (Zhong et al., 2007) and AtMYB83 (McCarthy et al., 2009) are direct target genes of AtSND1, and the induction of these two TFs in turn stimulates the expression of downstream lignin biosynthesis genes, including *PAL1*, *C4H*, *4CL1*, *CCoAOMT*, *HCT*, *CCR1*, and *F5H1* (Ko et al., 2009; McCarthy et al., 2009; Zhong and Ye, 2011; Kim et al., 2012; Kim et al., 2014). In addition, several MYB genes are also involved in the regulation of lignin biosynthesis, including AtMYB20, AtMYB42, AtMYB43, AtMYB58, AtMYB63, and AtMYB85 (Zhou et al., 2009; Geng et al., 2019).

In *Citrus sinensis*, the CsMYB330 and CsMYB308 act as transcriptional activator and repressor of fruit juice sac lignification by directly interacting with *Cs4CL1* (Jia et al., 2018). Transcription factor CsMYB85 has been reported to interact with CsMYB308 and bind to the promoter of CsMYB330, thus regulating the expression of *Cs4CL1* and fruit juice sacs lignification (Jia et al., 2019). In *Citrus grandis*, CgMYB58 (homolog of CsMYB85) has been reported to regulate the expression of *CgPAL1*, *CgPAL2*, *Cg4CL1*, and *CgC3H* during fruit juice sac granulation through the direct interaction with the AC elements in their promoters (Shi et al., 2020). However, all these transcription factors have been identified in the post-harvest induced granulation, the transcription factors involved in the cold-induced granulation remain very limited.

In this study, a typical SG2-type R2R3 MYB transcription factor, CsMYB15, was identified and characterized in *Citrus sinensis.* The expression of *CsMYB15* was up-regulated under different granulation degrees and induced by low temperature treatment in navel orange fruits. Subcellular localization analysis showed that CsMYB15 protein was localized in the nucleus and had transcriptional activation activity in yeast. Over-expression of *CsMYB15* increased lignin content in juice sacs, kumquat fruits, and navel orange calli. CsMYB15 bound to the *Cs4CL2* promoter to regulate its expression, thus mediating lignin biosynthesis, which indicated that CsMYB15 was involved in the low temperature-induced juice sac granulation progress. This study not only provides a comprehensive analysis of the biological function and regulating mechanism of *CsMYB15*, but also sheds novel insight into the transcriptional regulation network of lignin biosynthesis in low temperature-induced fruit juice sac granulation process in ‘Lane late’ navel orange.

## 2 Materials and methods

### 2.1 Plant materials

A total of 9 mature ‘Lane late’ navel orange (*Citrus sinensis* Osbeck) trees (10-year-old) with red tangerine (*Citrus tangerina* Hort) as rootstock from an orchard located in the Three Gorges Reservoir area (E 110°41’, N 30°54’) in Zigui County were selected for sampling in this study, and fruits were collected at 350 days after flowering according to our previous study (Wu et al., 2020). The experiments were conducted with three biological replicates, and 15 fruits collected from 3 trees in different directions were mixed together as one biological replicate. The juice sacs were isolated from the fruits, immediately frozen with liquid nitrogen, and stored at -80 °C for RNA extraction. For low temperature treatment, the juice sacs isolated from normal ‘Lane late’ navel orange fruits were placed on a plate and covered with sterilized gauze to keep moist. Then, the plates with juice sacs were transported into an incubator at 4 °C for low temperature treatment. The samples were collected at 0 h, 3 h, 9 h, 12 h, and 24 h, and 30 individual juice sacs were collected as one biological replicate. Each sample had three biological replicates, and all the juice sacs were immediately frozen with liquid nitrogen and stored in -80 °C for RNA extraction.

### 2.2 Phylogenetic analysis

To explore the phylogenetic relationship of CsMYB15 and other MYB family transcription factors, a multiple sequence alignment of the amino acid sequences of CsMYB15 and other 32 MYB proteins was performed using MUSCLE (www.ebi.ac.uk/Tools/msa/muscle/ ) with default parameters (Edgar, 2004). The phylogenetic tree was constructed using the maximum likelihood method of MEGA X software with 1000 bootstraps (Kumar et al., 2018). The final phylogenetic tree was visualized and polished with the Interactive Tree of Life software (iTOL, version 5, https://itol.embl.de/) (Letunic and Bork, 2016).

### 2.3 RNA extraction, cDNA synthesis and qRT-PCR

Total RNA was extracted from the juice sacs using TRIzol™ reagent according to the manufacturer’s instructions (Thermo Scientific). RNA quality was assessed by agarose gel electrophoresis, and 5X All-In-One RT MasterMix with AccuRT (Applied Biological Materials) kit was used for ss/dsDNA digestion and cDNA synthesis. Quantitative RT-PCR was performed on a QuantStudio 7 Flex system (Thermo Scientific, USA) according to the manufacturer’s instruction with EvaGreen 2X qPCR MasterMix kit (Applied Biological Materials, Canada). *Elongation factor 1* (*Ef1*, Cs8g16990) gene was utilized as an internal reference gene (Li et al., 2022), and the relative expression was calculated with 2^-ΔΔCt^ method. Student’s *t*-test was performed to determine statistical significance, and ** indicated significant differences at the level of *P* < 0.01. The primers used for qRT-PCR were listed in **Supplementary Table S1**.

### 2.4 Cloning of *CsMYB15* gene and promoter

The cDNA synthesized from the total RNA extracted from juice sacs of ‘Lane late’ navel orange (*Citrus sinensis* Osbeck) was used to amplify the full-length coding sequence (CDS) of *CsMYB15* using the following primers: Forward 5’ -ATGATGGGGAGGGCTCC- 3’ and Reverse 5’ -GGAAATGGTAATGTTAATGAGTCTGCC-3’. PCR amplification was performed using the Phanta Max Super-Fidelity DNA Polymerase (Vazyme, Nanjing, China) following the manufacturer’s instructions. The PCR product was purified and cloned into pTOPO vector for sequencing. The coding sequence of *CsMYB15* was deposited in Genebank with the accession number of OQ985393. The 2000 bp region upstream the start codon ATG of *CsMYB15* was cloned as the promoter sequence from the total DNA extracted from the leaf of ‘Lane late’ navel orange. The promoter of *CsMYB15* was further analyzed using the online software of plantPan 3.0 (http://plantpan.itps.ncku.edu.tw/) and plantCARE (http://bioinformatics.psb.ugent.be/webtools/plantcare/html/). The promoter sequence of *CsMYB15* was provided in **Supplementary Table S2**.

### 2.5 Subcellular localization analysis of CsMYB15

The CDS of *CsMYB15* without stop codon was ligated with GFP (green fluorescent protein), and then cloned into pICH86988 vector containing cauliflower mosaic virus 35S promoter to construct 35S:CsMYB15:GFP vector by Golden Gate method (Engler et al., 2014). Subsequently, the recombinant 35S:CsMYB15:GFP vector was transformed into *Agrobacterium tumefaciens* (strain GV3101 with pSoup-p19), and co-expressed with a nucleolus marker 35S:FIB2:mCherry in tobacco leaves (Chang et al., 2016) according to a previous study (Sparkes et al., 2006). The subcellular localization of CsMYB15 was observed via a confocal laser-scanning microscope (TCS SP6, Leica, Germany).

### 2.6 Validation of CsMYB15 transcriptional activity

A yeast system was utilized to examine the transcriptional activity of CsMYB15 according to a previously described method (Ma et al., 2009). The coding sequences of *CsMYB15* were cloned into the bait vector pGBKT7 by homologous recombination method using HiFi DNA Assembly Master Mix (NEB, Ipswich, USA) according to the manufacturer’s protocol. The CsMYB15-pGBKT7 + pGADT7-AD, pGBKT7-lam + pGADT7-T (negative control), and pGBKT7-53 + pGADT7-T (positive control) were transformed into Y2H strain yeast independently. After being cultured on SD/-Trp medium at 30 °C for 3 days, single colonies were selected and further amplified. Subsequently, the positive transformants were grown on SD/-Leu/-Trp and SD/-Leu/-Trp/-His/-Ade media, and the growth of all the transformants were observed after 3 days. Additionally, the galactosidase assay was performed by adding X-α-Gal to the medium according to a previously described method (Ma et al., 2009).

### 2.7 Transformation of *CsMYB15* in juice sacs, kumquat fruits, and citrus calli

The CDS of *CsMYB15* was cloned into pAML4 vector (containing cauliflower mosaic virus 35S promoter-driven GFP reporter module) together with CaMV35S promoter and nos terminator through Golden Gate method (Engler et al., 2014). The recombinant plasmid was transformed into *Agrobacterium tumefaciens* (strain GV3101) for further transformation. For juice sac transient expression, juice sacs were separated from ‘Lane late’ navel orange (*Citrus sinensis* Osbeck) fruits, and *CsMYB15* was transiently over-expressed in juice sacs, as described in previous study (Shi et al., 2019). The transformed juice sacs were carefully collected at 6 days post infection. For kumquat (*F. crassifolia* Swingle) fruit transient expression, kumquat fruits at the green ripening stage (120-180 days after flowering) were selected for transformation. These fruits were infiltrated with *A. tumefaciens* carrying the *CsMYB15* expression vector by using a sterile 1 ml hypodermic syringe according to the method described previously (Gong et al., 2021a). The infiltrated fruit sections were screened with GFP fluorescence and sampled at 5 days post infection by using a fluorescence microscope (Olympus, Japan). For citrus calli stable transformation, the healthy and plump calli of navel orange (*Citrus sinensis* Osbeck) were selected, and the transformation was performed according to a previously described method (Shi et al., 2020). The positive calli were selected by GFP fluorescence using a fluorescence microscope (Olympus, Japan), and then validated by PCR amplification.

Half of the juice sac, kumquat fruit, and citrus callus samples were immediately frozen with liquid nitrogen and stored at −80°C for RNA extraction and qRT-PCR analysis. The remaining samples were stoved in a bake oven for lignin content determination.

### 2.8 Determination of lignin contents

The lignin contents of the juice sacs, kumquat fruits, and citrus calli were measured by using a lignin assay kit (Solarbio, China) according to the manufacturer’s instruction (Yang et al., 2022), and each sample has three biological replicates.

### 2.9 Yeast one-hybrid assay (Y1H)

Yeast one-hybrid assay was conducted as described previously (Gong et al., 2021b). The full-length CDS of CsMYB15 and promoter sequences of *Cs4CL1*, *Cs4CL2*, *CsC3H*, *CsCCR2*, *CsPOD2*, and *CsPOD3* were cloned into pGADT7 and pAbAi vectors to produce prey construct of pGADT7:CsMYB15 and bait constructs of pAbAi:promoters, respectively. Then, the bait constructs were transformed into the yeast strain Y1H Gold to produce reporter strains, and their autoactivation was determined on SD/-Ura+ aureobasidin A (AbA) plates. The prey construct was introduced into the reporter strains, and pGADT7 empty vector was utilized as a negative control. The transformed yeast cells were cultured on SD/-Ura/-Leu/AbA medium for Y1H assay to examine the interaction between CsMYB15 and the promoters of *Cs4CL1*, *Cs4CL2*, *CsC3H*, *CsCCR2*, *CsPOD2* and *CsPOD3*. The primers used for constructing Y1H vectors were listed in **Supplementary Table S1**. The promoter sequences of *Cs4CL1*, *Cs4CL2*, *CsC3H*, *CsCCR2*, *CsPOD2* and *CsPOD3* were deposited in Genebank with the accession number of OQ985394 to OQ985399.

### 2.10 Electrophoretic mobility shift assay (EMSA)

The electrophoretic mobility shift assay (EMSA) was conducted as described previously with minor modifications (Shi et al., 2020). Briefly, the MBP-CsMYB15 protein was expressed and purified, as described previously (Lu et al., 2018). The 5’ FAM-labeled oligonucleotide probes were directly synthesized and labeled by the Tianyi Huiyuan Company (Wuhan, China). The same oligonucleotides without labels were utilized as competitors. Probe information was provided in **Supplementary Table S1**.

### 2.11 Dual luciferase transcriptional activity assay (LUC)

Dual luciferase transcriptional activity assay was performed using *Nicotiana benthamiana* leaves according to a previous report (Lu et al., 2018). Specifically, the the promoter sequence of *Cs4CL2* was inserted into the upstream of LUC coding sequence in pGreen0800-LUC vector to produce reporter construct. The full-length CDS of CsMYB15 was cloned into pAML4 over-expression vector to produce 35S::CsMYB15 effector construct. The effector construct and reporter construct were respectively transformed into *A. tumefaciens* (strain GV3101 with pSoup-p19), and co-expressed in tobacco leaves according to a previously reported method (Hellens et al., 2005). The LUC activity was measured according to the method reported by Lu et al. (2018). All the primers were listed in **Supplementary Table S1**.

## 3 Results

### 3.1 Cloning and characterization of *CsMYB15* in *Citrus sinensis*

**Figure 1:**
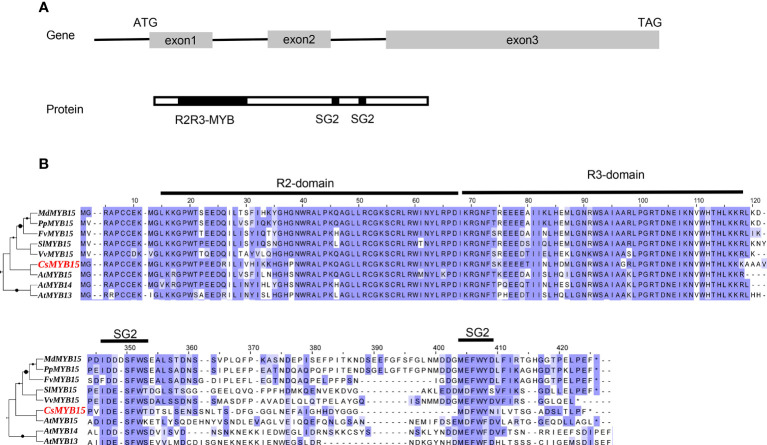
Schematic diagram and amino acid sequence alignment of *CsMYB15*. **(A)**. Schematic diagram of the *CsMYB15* gene (top) and protein (bottom). **(B)**. Multiple sequence alignment of CsMYB15 amino acid sequence with homologous MYBs in other species. R2, R3, and SG2 domains were marked with dark lines.

The coding sequence of *CsMYB15* was identified and cloned from ‘Lane late’ navel orange (*Citrus sinensis* Osbeck) to investigate its biological function. The coding sequence of *CsMYB15* was 804 bp in length, and this coding sequence encoded a 268-amino acid protein. As shown in **Figure 1A**, the open reading frame of *CsMYB15* was constructed with three exons. Then, the coding sequence of *CsMYB15* was aligned with that of 8 other MYB genes with high homology. CsMYB15 protein was found to contain four conserved domains, including one R2-domain, one R3-domain, and two SG2 domains, indicating that CsMYB15 was an typical SG2-Type R2R3-MYB protein (**Figure 1B**).

### 3.2 Phylogenetic analysis of *CsMYB15*

**Figure 2:**
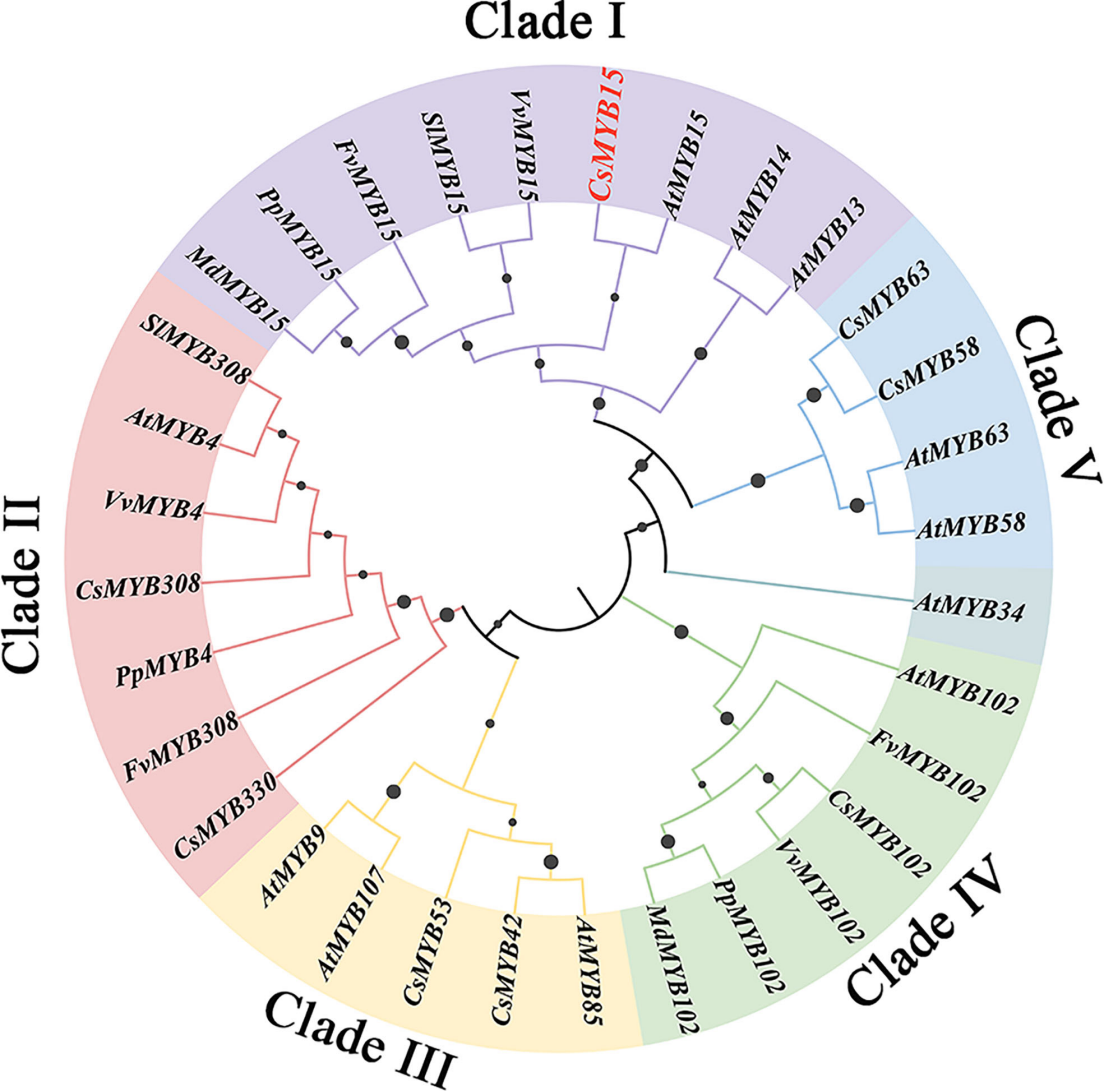
Phylogenetic analysis of MYB15-related proteins and lignin biosynthesis-related MYBs in different plant species. At, *Arabidopsis thaliana*; Vv, *Vitis vinifera*; Sl, *Solanum lycopersicum*; Fv, *Fragaria vesca*; Pp, *Prunus persica*; and Md, *Malus domestica*. CsMYB15 (CS00088G00320 or orange1.1g024484m), FvMYB4 (FV4G00360.1), MdMYB15 (MD00G513530), PpMYB15 (PPE_001G03850), SlMYB15 (SL09G090130), VvMYB15 (VV05G08250), AtMYB14 (AT2G31180), AtMYB13 (AT1G06180), AtMYB107 (AT3G02940), CsMYB102 (CS00010G02180), AtMYB102 (AT4G21440), PpMYB102 (PPE_004G03340), FvMYB39 (FV3G07360), MdMYB102 (MD00G267480), VvMYB102 (VV00G07100), CsMYB9 (CS00312G00030), AtMYB9 (AT5G16770), AtMYB63 (AT1G79180), AtMYB58 (AT1G16490), AtMYB34 (AT5G60890), AtMYB4 (AT4G38620), CsMYB4 (CS00023G00610), PpMYB4 (PPE_008G18180), FvMYB308 (FV2G07290), VvMYB4 (VV03G04830), SlMYB308 (SL01G111500), AtMYB85 (AT4G22680), CsMYB308 (CS00271G00230), CsMYB330 (CS00002G01660), CsMYB58 (CS00052G00740).

To explore the phylogenetic relationship of *CsMYB15*, a phylogenetic tree was established with the amino acid sequences of 32 MYB proteins using the maximum likelihood method of MEGA. As shown in **Figure 2**, the MYB genes were divided into 5 clades, including Clade I, Clade II, Clade III, Clade IV and Clade V, and the *CsMYB15* was highly homologous to MYB15-like genes from *Arabidopsis thaliana*, *Vitis vinifera*, *Solanum lycopersicum*, *Fragaria vesca*, *Prunus persica*, and *Malus domestica* in Clade I. It was worth noting that *CsMYB15* and *AtMYB15* were clustered into a subclade with the closest genetic relationship and the highest homology. Further blast result revealed that *CsMYB15* was the orthologous gene of cold tolerance-related *AtMYB15*.

### 3.3 Expression profiles of *CsMYB15*

**Figure 3:**
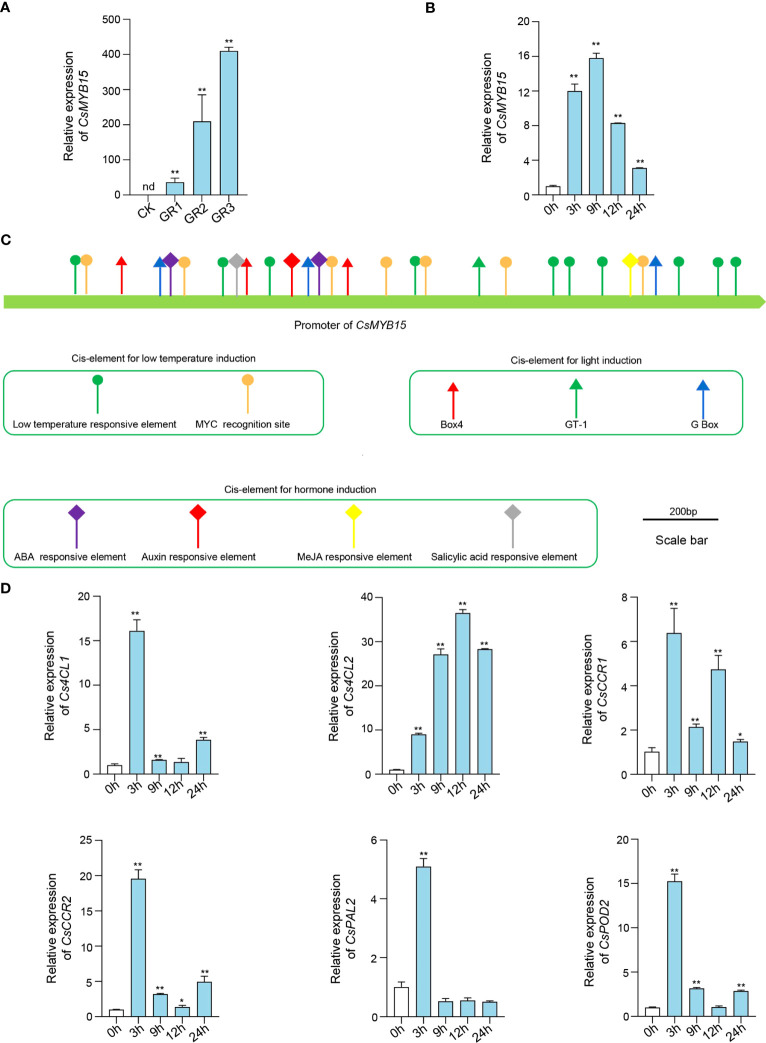
Expression profiles of *CsMYB15* under different juice sac granulation stages and low temperature treatment. **(A)**. qRT-PCR analysis of transcript abundance of *CsMYB15* in ‘Lane late’ navel orange fruits under different juice sac granulation stages. CK, non-granulated fruit; GR1, slight granulated fruit; GR2, moderate granulated fruit; GR3, serious granulated fruit; nd, not detected. **(B)**. qRT-PCR analysis of the relative expression of *CsMYB15* in the fruits treated with low temperature. **(C)**. Schematic diagram of the *CsMYB15* promoter in ‘Lane late’ navel orange. **(D)**. qRT-PCR analysis of the relative expression of lignin biosynthesis genes in the fruit juice sacs treated with low temperature.*CsEF1* was used as an internal control. All data were expressed as mean ± SE. **, *P* < 0.01, *, *P* < 0.05 (student’s *t*-test).

To further investigate the role of *CsMYB15* in low temperature-induced juice sac granulation, the expression of *CsMYB15* under different degrees of low temperature-induced juice sac granulation was assessed by qRT-PCR. As shown in **Figure 3A**, the expression of *CsMYB15* was significantly induced by juice sac granulation, and the induction of *CsMYB15* was gradually increased with the increasing granulation degrees, indicating that *CsMYB15* might be involved in the regulation network during juice sac granulation process. Interestingly, we also found that the expression of *CsMYB15* was significantly induced under low temperature treatment (**Figure 3B**). Although the expression of *CsMYB15* was increased first and then decreased under low temperature treatment, it was relatively higher at all treatment time points than at 0 h, suggesting a significant induction of *CsMYB15* under low temperature stress. Further, we amplified the promoter sequence of *CsMYB15* from ‘lane late’ navel orange, and analyzed the *cis*-elements involved in the promoter sequence. As shown in **Figure 3C**, the potential regulatory *cis*-elements were predicted, including low temperature responsive element, MYC recognition site, which have been predicted to be involved in low temperature responsiveness in many cases. We also predicted the light responsiveness elements, Box 4, GT-1, and G Box, and hormone responsiveness elements from the promoter of *CsMYB15*, indicating that the expression level of *CsMYB15* might also been regulated by light and plant hormones. Additionally, qRT-PCR revealed that the expression level of 6 lignin biosynthesis genes were up-regulated under low temperature treatment (**Figure 3D**). Expect for the expression level of *CsPAL2* was only induced at 3h, the expression levels of *Cs4CL1*, *Cs4CL2*, *CsCCR1*, *CsCCR2*, and *CsPOD2* were increased first and then decreased, and the expression levels were relatively higher at all treatment time points than at 0 h, which was in line with the expression level of *CsMYB15*. In a word, these results indicating that *CsMYB15* was induced by both juice sac granulation and low temperature treatment.

### 3.4 Subcellular localization and transcriptional activity of CsMYB15

**Figure 4:**
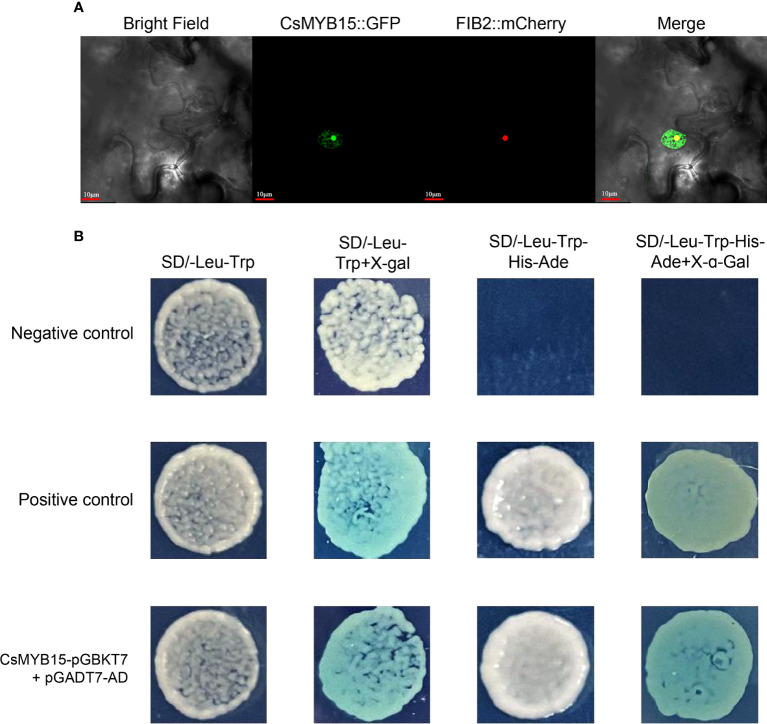
Subcellular localization and transcriptional activity of CsMYB15. **(A)**. Subcellular localization of CsMYB15 in *N. benthamiana* leaves. Scale bars = 10 μm. **(B)**. Transcription activity analysis of CsMYB15 protein in yeast. pGBKT7-lam + pGADT7-T were co-transformed into yeast as the negative control, and pGBKT7-53 + pGADT7-T were co-transformed into yeast as the positive control.

To explore the subcellular localization of CsMYB15 protein, CsMYB15-GFP fusion construct in pICH86988 vector was transiently co-expressed with a nucleolus marker (FIB2:mCherry) in tobacco leaves. As shown in **Figure 4A**, the green fluorescence of CsMYB15:GFP was localized to the nucleus, including both nucleolus and nucleoplasm. The GFP fluorescence of nucleolus was overlapped with the red fluorescence of FIB2:mCherry (a nucleolus marker), and the nucleolus was surrounded by the GFP fluorescence of nucleoplasm. A yeast system was adopted for assessing the transcriptional activity of CsMYB15. As shown in **Figure 4B**, the positive control and pGBKT7-CsMYB15 strains grew well on both SD/-Leu-Trp and SD/-Leu-Trp-His-Ade media, and these two strains turned blue on the medium containing X-α-gal, whereas the negative control strain failed to grow on SD/-Leu-Trp-His-Ade medium, indicating that CsMYB15 had transcriptional activation activity in yeast. Taken together, the above results suggested that CsMYB15 was a nuclear-localized protein with transcriptional activation activity.

### 3.5 Over-expression of CsMYB15 increases lignin content in transgenic plants

**Figure 5:**
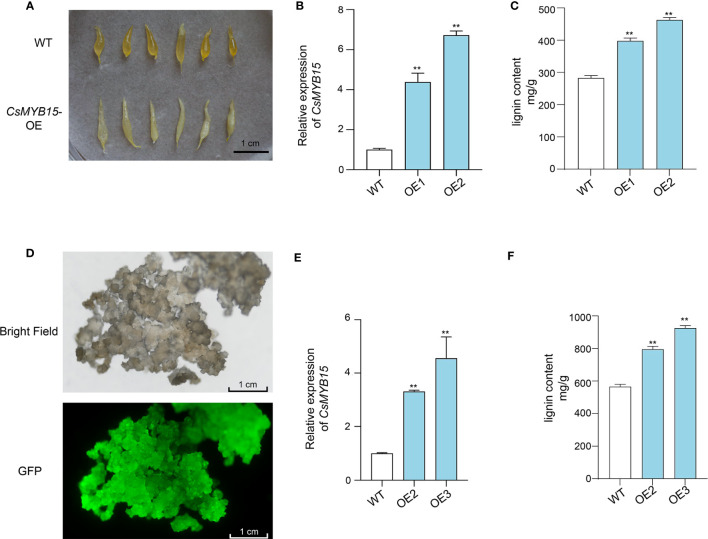
Transient expression of *CsMYB15* in navel orange juice sacs and stable expression of *CsMYB15* in navel orange calli. **(A)**. The morphology of transgenic juice sacs over-expressing *CsMYB15* was slim, deformed, and granulated. **(B)**. Relative expression levels of *CsMYB15* in WT and two transgenic juice sac lines over-expressing *CsMYB15*. **(C)**. Lignin content in WT and two transgenic juice sac lines over-expressing *CsMYB15*. **(D)**. Positive citrus calli over-expressing *CsMYB15* under bright field and GFP fluorescence. **(E)**. Relative expression level of *CsMYB15* in WT and two transgenic citrus callus lines over-expressing *CsMYB15*. **(F)**. Lignin content in WT and two transgenic citrus callus lines over-expressing *CsMYB15*. All data were expressed as mean ± SE. The asterisks indicate significant differences according to student’s *t*-test (** *P* < 0.01).

The coding sequence of *CsMYB15* was cloned from ‘Lane late’ navel orange, and individual transgenic experiments were conducted to explore the biological function of *CsMYB15*. Firstly, *CsMYB15* was transiently expressed in the juice sacs isolated from ‘Lane late’ navel orange fruits. As shown in **Figure 5B**, qRT-PCR revealed that the expression level of *CsMYB15* was up-regulated in the juice sac over-expressing *CsMYB15*. Comparing with that of wild type, the morphology of juice sacs transiently expressing *CsMYB15* was slim, deformed, and granulated (**Figure 5A**). The lignin content in juice sacs over-expressing *CsMYB15* was significantly higher than that in wide type (**Figure 5C**). These results were further verified by the transient expression of *CsMYB15* in ‘Huapi’ kumquat (*Fortunella crassifolia* Swingle) fruits (**Figure S1**).

Then, *CsMYB15* was over-expressed in citrus calli through *Agrobacterium*-mediated stable transformation system. Two independent transgenic lines were selected through GFP fluorescence (**Figure 5D**). The expression of *CsMYB15* in representative lines was validated by qRT-PCR (**Figure 5E**). As expected, the lignin content was significantly higher in the transgenic citrus calli over-expressing *CsMYB15* than in wide type calli (**Figure 5F**). Taken together, these results indicated that *CsMYB15* positively regulated lignin biosynthesis in citrus.

### 3.6 CsMYB15 activates the expression of *Cs4CL2* by directly binding to its promoter

**Figure 6:**
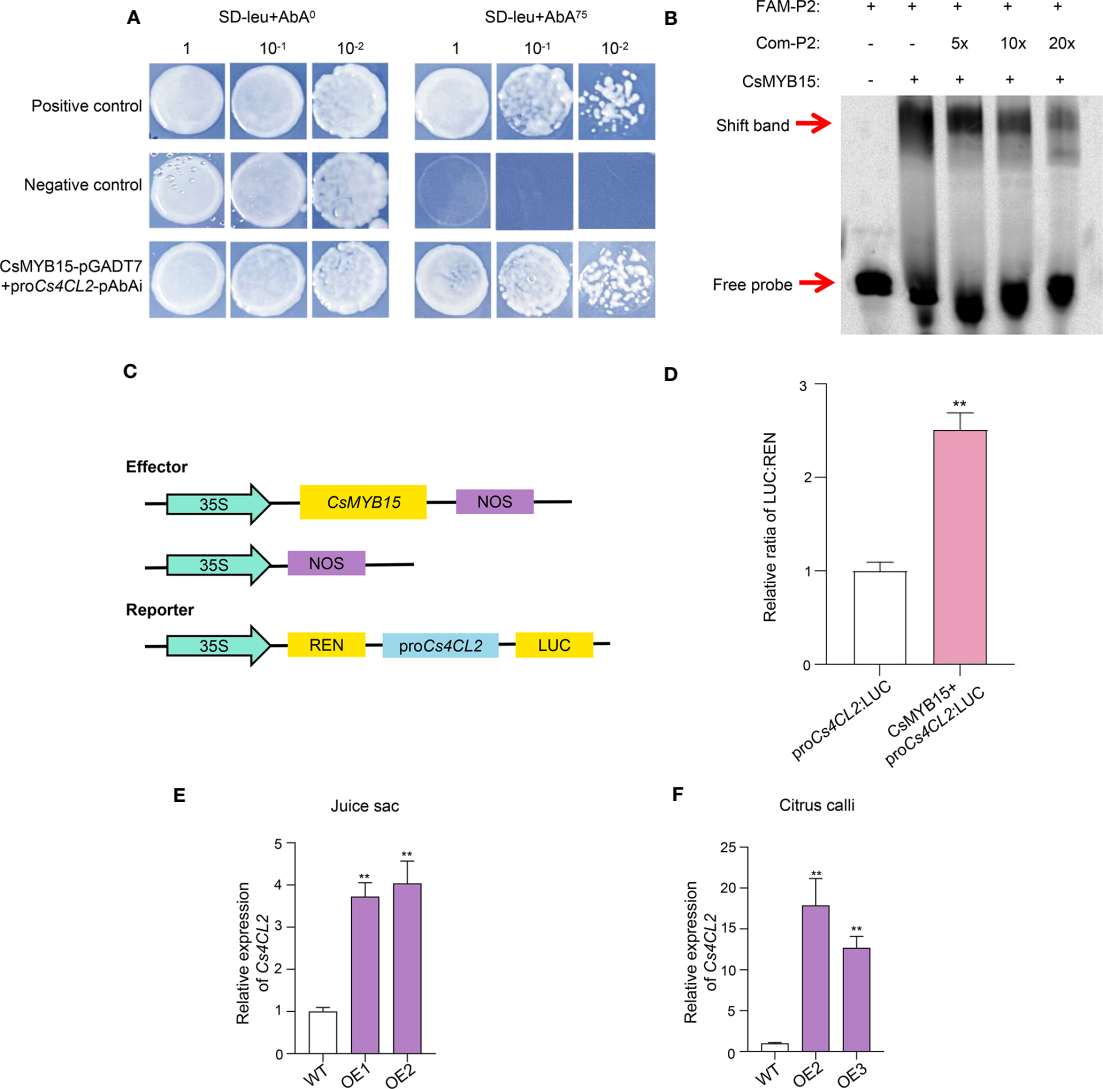
CsMYB15 directly binds to and transactivates the promoter of *Cs4CL2*. **(A)** CsMYB15 could interact with the promoter of Cs4CL2 in yeast one-hybrid assay. **(B)** CsMYB15 directly bound to the MRS elements in the promoter of Cs4CL2 according to EMSA assay. **(C)** Schematic diagram of effector and reporter structures used for dual luciferase assay. LUC, firefly luciferase; REN, Renilla luciferase. **(D)** CsMYB15 increased the activity of the Cs4CL2 promoter according to the LUC assay. **(E)** Relative expression levels of Cs4CL2 in WT and two transgenic juice sac lines over-expressing CsMYB15. **(F)** Relative expression levels of Cs4CL2 in WT and two transgenic citrus callus lines over-expressing CsMYB15. The asterisks indicate significant differences according to student’s t-test (** *P* < 0.01).

We speculated that CsMYB15 could directly bind to the promoters of lignin biosynthesis genes, thus affecting their expression, eventually regulating the lignin biosynthesis. Thus, 2000 bp sequences upstream the initiation codon were cloned as the promoters of *Cs4CL1*, *Cs4CL2*, *CsC3H*, *CsCCR2*, *CsPOD2* and *CsPOD3*, and the Yeast one-hybrid assay (Y1H) was conducted to verify the interaction between CsMYB15 and the promoters of these genes. As shown in **Figure 6A**, clone CsMYB15 + pro*Cs4CL2* was grown well on the SD-leu + 75 ng/μL AbAi (aureobasidin A) medium, indicating that CsMYB15 could bind to the promoter of *Cs4CL2.* However, CsMYB15 did not interact with the promoters of *Cs4CL1* (**Figure S2**), it did not interact with those of *CsC3H*, *CsCCR2*, *CsPOD2* and *CsPOD3*, either (data not shown).

We predicted that CsMYB15 might bind to the MYB recognition sites (MRS, TGGTTG/A) on the *Cs4CL2* promoter. To verify our prediction, the electrophoretic mobility shift assay (EMSA) was performed using purified MBP-CsMYB15 protein with a FAM-labeled *Cs4CL2* promoter fragment (−51 to −89 bp region) used as a probe. As shown in **Figure 6B**, a shifted band was observed after the probe was incubated with MBP-MYB15, but this band became weaker under the actions of the corresponding competitors in a dosage-dependent manner. These results further demonstrated that CsMYB15 directly bound to the MRS cis-element of *Cs4CL2* promoter *in vitro*.

In addition, a dual luciferase transcriptional activity assay (LUC) was performed in *N. benthamiana* leaf to reveal how CsMYB15 regulated the expression of *Cs4CL2.* The LUC: renillia (REN) ratio of leaf co-expressing CsMYB15 and *Cs4CL2* promoter was significantly higher than that of the negative control, indicating that CsMYB15 acted as transcriptional activators of *Cs4CL2* by directly interacting with the MRS elements (**Figures 6C, D**). Further, we detected the expression patterns of *Cs4CL2* in the transgenic juice sac and citrus callus lines using qRT-PCR. As shown in **Figures 6E, F**, the expression level of *Cs4CL2* was significantly higher in the transgenic plant lines over-expressing *CsMYB15* than the wide type, which was further proof of the activation of CsMYB15 transcription factor on the *Cs4CL2.*

## 4 Discussion

Juice sac granulation is a severe physiological disorder in citrus fruit, causing dramatic decline in fruit quality and commercial value (Shi et al., 2020; Wu et al., 2020). In recent years, extensive studies have been conducted to elucidate the physiological and molecular mechanisms underlying the juice sac granulation (Wu et al., 2014; Yao et al., 2018; Jia et al., 2019; Shi et al., 2020; Zhang et al., 2021). However, most of existing studies focus on post-harvest juice sac granulation, the molecular regulating network of low temperature-induced pre-harvest juice sac granulation remains largely unknown. In this study, we identified SG2-type R2R3-MYB transcription factor *CsMYB15* as a positive regulator of lignin biosynthesis, and *CsMYB15* could directly bind to the *Cs4CL2* promoter to activate its transcription during low temperature-induced juice sac granulation in late-maturing navel orange.

Low temperature is one of the major abiotic stress limiting the plant growth, development, quality, and yield (Ma et al., 2015; Ding et al., 2020). In horticultural plants, low temperature usually causes granulation of the chilling sensitive fruits such as loquat (Cai et al., 2006; Zhang et al., 2020a), kiwifruit (Suo et al., 2018), pear (Lu et al., 2014), and zucchini (Carvajal et al., 2015). Our previous study has shown that low temperature in winter induced pre-harvest juice sac granulation in ‘Lane late’ navel orange by affecting the cell wall metabolism and increasing the accumulation of lignin (Wu et al., 2020). MYB transcription factors play critical roles in plant stress responses, especially abiotic stress responses including cold (Agarwal et al., 2006; An et al., 2018), drought (Nakabayashi et al., 2013; Zhao et al., 2018), and salt stress responses (Sun et al., 2020; Zhang et al., 2020c). Here, *CsMYB15* was identified as a typical SG2-type R2R3 MYB transcription factor in *Citrus sinensis*. Our phylogenetic analysis revealed that *CsMYB15* was the orthologous gene of *AtMYB15.* In agreement with previous report that plants exhibited increased accumulation of MYB15 transcript in response to cold stress in both Arabidopsis (Agarwal et al., 2006) and tomato (Zhang et al., 2020b), we also found that *CsMYB15* expression was up-regulated under low temperature treatment in navel orange fruits, and *CsMYB15* was highly expressed in the granulated navel orange fruits, and its expression level was increased with the increasing severity of juice sac granulation. Additionally, CsMYB15 protein was localized to the nucleus and had transcriptional activation activity in yeast. Thus, *CsMYB15* might act as a low temperature-induced transcriptional activator involved in juice sac granulation.

MYB TFs play important roles in regulating lignin biosynthesis in plants (Taylor-Teeples et al., 2014; Liu et al., 2015; Chen et al., 2019; Xiao et al., 2021). In *Pinus taeda*, *PtMYB4* can induce lignication during wood formation (Patzlaff et al., 2003). In *Populus trichocarpa*, over-expression of *PtrMYB3* and *PtrMYB20*, and *PtoMYB216* activates the expression of the upstream genes in the lignin biosynthetic pathway, thus resulting in the lignin deposition. In *Arabidopsis thaliana*, *AtMYB58*, *AtMYB63*, and *AtMYB85* regulate the lignification in vascular tissues by binding to the AC-rich elements in the promoters of lignin biosynthesis genes (Zhong et al., 2008; Zhou et al., 2009). *AtMYB15*, a homologous gene of *CsMYB15*, increases the biosynthesis of G-lignin to promote defence-induced lignification and basal immunity under pathogen infection (Chezem et al., 2017; Kim et al., 2020). The present study showed that over-expression of *CsMYB15* led to an increase in the lignin content in all the transgenic juice sacs, kumquat fruits, and citrus calli, indicating that *CsMYB15* positively regulated lignin biosynthesis in citrus. Our Y1H, EMSA, and LUC assays further demonstrated that CsMYB15 could bind directly to the MRS element in the promoter of *Cs4CL2* to activate its expression. However, CsMYB15 failed to bind to the MRS element in the promoter of *Cs4CL1*, indicating that CsMYB15 might not bind to all the promoters containing MRS element. Similar results have also been reported in Arabidopsis and Citrus. AtMYB58 and AtMYB63 bound to and activated the promoter of *AtLAC4*, but did not bind to the promoter of *AtLAC17* (Zhou et al., 2009)*. CsMYB330* and *CsMYB308* bound to AC elements in the *Cs4CL1* promoter, but not to the promoters of *CsCCoAOMT1*, *CsPAL1*, and *CsPAL2* although these promoters also contained AC elements (Jia et al., 2018).

Previous studies have shown that the lignin biosynthesis pathway is centrally involved in the juice sac granulation, and four R2R3-MYB transcription factors have been identified to be involved in the post-harvest juice sac granulation process in sweet orange and pummelo by regulating lignin biosynthesis, including CgMYB58, CsMYB85 (homolog of CgMYB58), CsMYB330, and CsMYB308. In this study, a novel SG2-type R2R3-MYB transcription factor, CsMYB15, was identified as a transcriptional activator of lignin biosynthetic gene *Cs4CL2*, and it could directly bind to its promoter, thereby resulting in the accumulation of lignin during low temperature-induced pre-harvest juice sac granulation. Our phylogenetic analysis demonstrated that *CsMYB15* was clearly separated from the reported four MYB transcription factors involved in the citrus juice sac granulation, and CsMYB15 was specifically induced by low temperature, indicating that *CsMYB15-*mediated lignin biosynthesis regulation network as a novel one was specific to the low temperature-induced pre-harvest juice sac granulation process.

**Figure 7:**
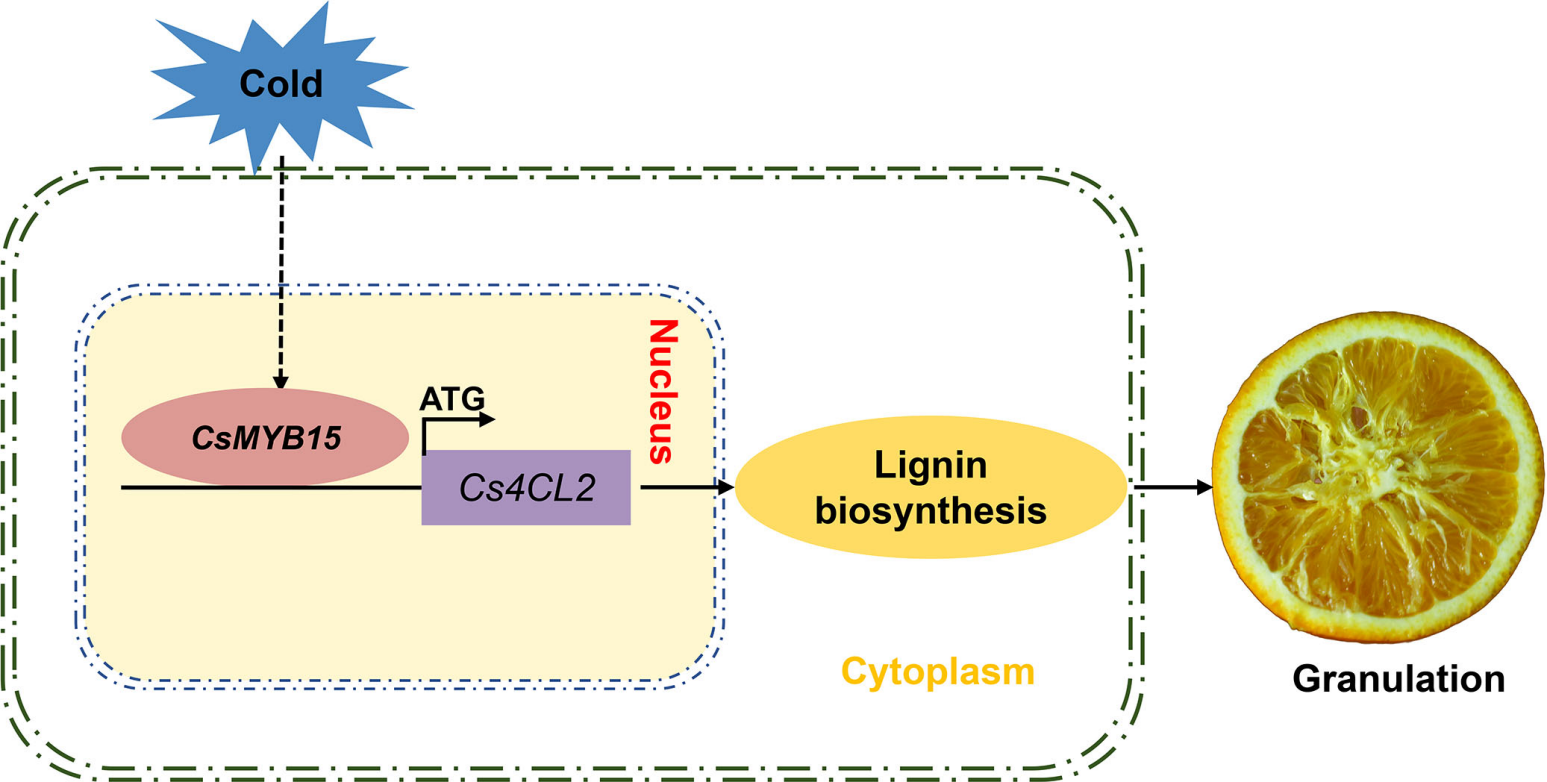
A proposed working model in which CsMYB15 regulates lignin biosynthesis during juice sac granulation in late-maturing navel orange. In this model, CsMYB15 is activated by cold stress in the winter, and then CsMYB15 directly binds to the promoter region of lignin biosynthesis gene *Cs4CL2* and induces its expression, leading to lignin biosynthesis, ultimately causing juice sac granulation in navel orange.

In conclusion, we proposed a working model in which CsMYB15 regulated lignin biosynthesis during low temperature-induced juice sac granulation process. CsMYB15 was activated by cold stress in the winter, and CsMYB15 transcriptionally activated the lignin biosynthetic gene *Cs4CL2* by directly binding to its promoter, thus promoting the accumulation of lignin in juice sacs, ultimately leading to the lignification of juice sacs in navel orange (**Figure 7**). Our findings advance our understanding of MYB transcriptional factor-mediated lignin biosynthesis regulation network during low temperature-induced pre-harvest juice sac granulation in late-maturing navel orange.

## Data availability statement

The original contributions presented in the study are included in the article/**Supplementary Material**. Further inquiries can be directed to the corresponding authors.

## Author contributions

FS, and LW conceived and designed the experiment. FS and ZL conducted the experiment and data analysis. YJ, CW, ZW, LH and XM contributed to the data analysis. FS and LW wrote the manuscript. YZ, XS and J-HL revised the manuscript. All authors contributed to the article and approved the final version.

## Supplementary material

Supplementary Figure 1 Transient expression of *CsMYB15* in kumquat fruits. **(A)**. Injection part of kumquat fruits. **(B)**. Relative expression levels of *CsMYB15* in WT and two transgenic kumquat fruit lines over-expressing *CsMYB15*. **(C)**. Lignin contents were measured in WT and two transgenic kumquat fruit lines over-expressing *CsMYB15*. All data were expressed as mean ± SE. The asterisks indicate significant differences according to student’s *t*-test (** *P* < 0.01).

Supplementary Figure 2 CsMYB15 does not interact with the promoter of *Cs4CL1* in yeast one-hybrid assay.
