# Supplementary figures and images for: CsMYB15 positively regulates Cs4CL2-mediated lignin biosynthesis during juice sac granulation in navel orange
Source: Front Plant Sci. 2023 Jun 30;14:1223820. doi: 10.3389/fpls.2023.1223820 (PMC10348809; doi:10.3389/fpls.2023.1223820)

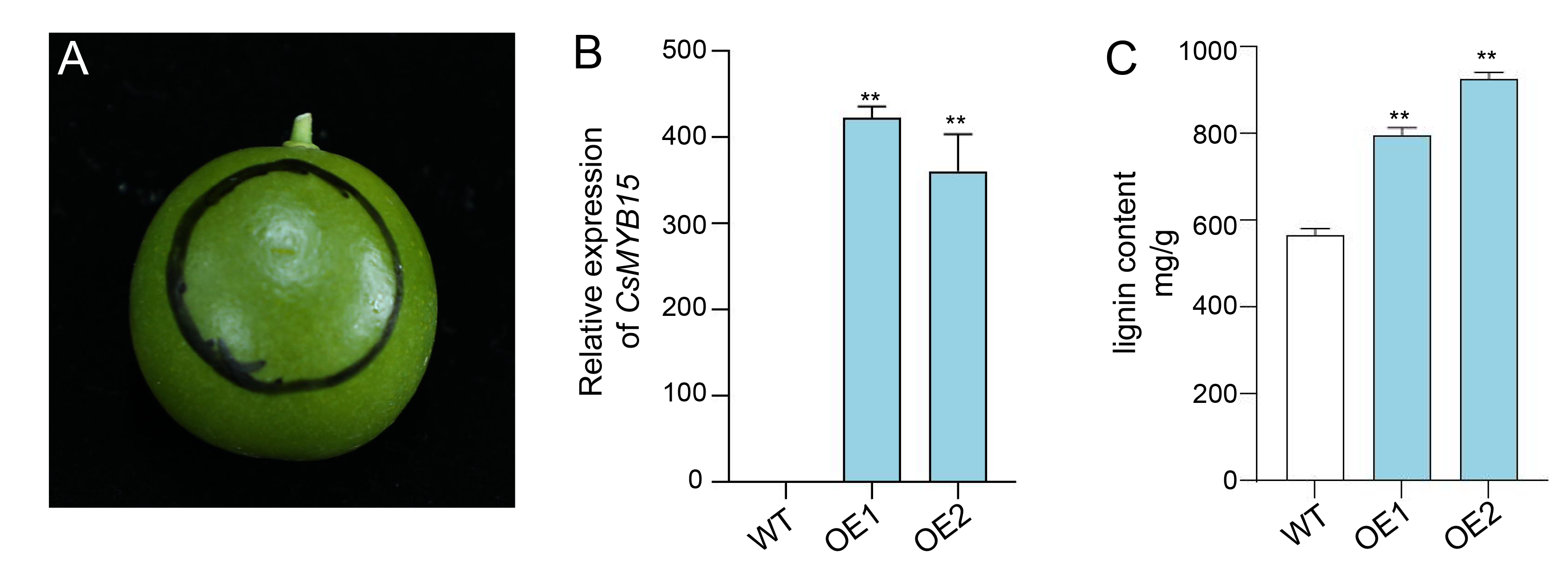

Supplement: Supplementary Figure 1 — Transient expression of CsMYB15 in kumquat fruits. (A). Injection part of kumquat fruits. (B). Relative expression levels of CsMYB15 in WT and two transgenic kumquat fruit lines over-expressing CsMYB15. (C). Lignin contents were measured in WT and two transgenic kumquat fruit lines over-expressing CsMYB15. All data were expressed as mean ± SE. The asterisks indicate significant differences according to student’s t-test (** P < 0.01). [file Image_1.tif]

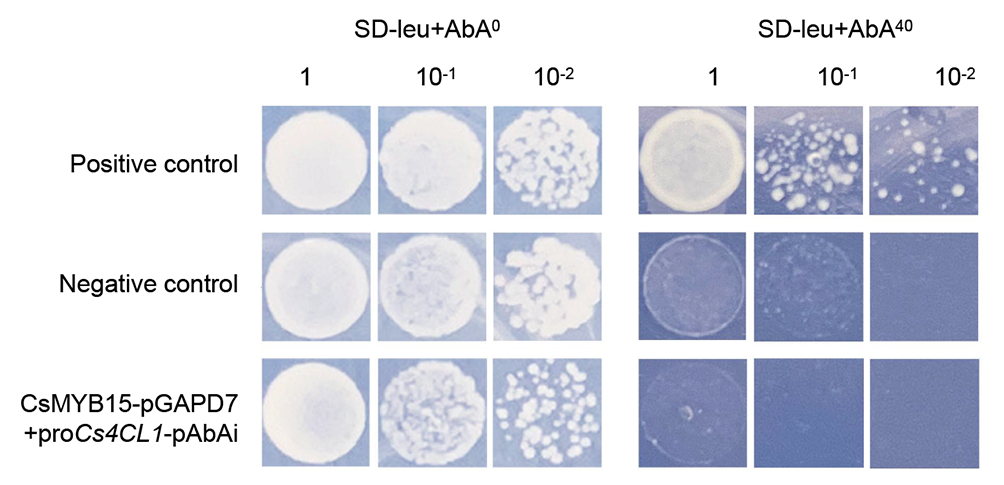

Supplement: Supplementary Figure 2 — CsMYB15 does not interact with the promoter of Cs4CL1 in yeast one-hybrid assay. [file Image_2.tif]
